# Burnout in emergency department staff: The prevalence and barriers to intervention

**DOI:** 10.4102/sajpsychiatry.v29i0.2095

**Published:** 2023-10-23

**Authors:** Reshen Naidoo, Renata Schoeman

**Affiliations:** 1 Faculty of Economics and Management Sciences, Stellenbosch Business School, Cape Town, South Africa

**Keywords:** burnout, prevalence, healthcare, hospital, emergency care, intervention, doctors, nurses, non-clinical

## Abstract

**Background:**

Burnout impacts patient care and staff well-being. Emergency department (ED) staff are at an elevated risk for burnout. Despite an acceleration in burnout research due to the coronavirus disease 2019 (COVID-19) pandemic, there is limited data on the nature and prevalence of burnout in the South African emergency medicine setting.

**Aim:**

This study determined the prevalence of burnout in ED staff (doctors, nurses and non-clinical staff) at Tygerberg Hospital and explored staff awareness and utilisation of interventions.

**Setting:**

The study was conducted at Tygerberg Hospital, South Africa.

**Methods:**

This cross-sectional study used the Maslach Burnout Inventory to assess burnout via a self-administered electronic survey in a convenience sample of 109 ED staff. Quantitative data were analysed with descriptive and inferential statistics. Qualitative data were analysed using thematic analysis.

**Results:**

A total of 46 participants (45.10%) experienced burnout, with 73 participants (71.57%) at high risk for emotional exhaustion or depersonalisation. The prevalence of burnout in doctors was 57.89%, non-clinical staff was 25.93%, and nursing staff was 50.00%. Burnout was higher in doctors and nursing staff compared to non-clinical staff, with high emotional exhaustion and depersonalisation found in interns and specialist professional nurses. The level of intervention awareness was 41.8% and the level of intervention utilisation was 8.82%. Thematic analysis identified awareness, accessibility and reactive utilisation as barriers to utilisation with opportunities to reduce burnout and enhance resilience.

**Conclusion:**

Coordinated health system and organisational efforts are required to optimise intervention strategies to reduce burnout.

**Contribution:**

Guidance on the design and planning of intervention strategies considering at risk groups, intervention-related factors, and non-clinical staff.

## Introduction

Burnout is a global concern in healthcare,^1^ adversely impacting the quality of care, patient satisfaction^2^ and staff well-being with an increased risk of substance abuse, medical errors, psychiatric disorders and suicide.^3,4,5^ Emergency department (ED) staff are at an elevated risk of burnout compared to other healthcare workers (HCW).^6,7,8^ The estimated prevalence in this subgroup is high globally, ranging from 49.3% to 58.0%,^9^ and is attributed to chronic workplace stressors.^10^

Multiple factors cause fatigue, anxiety and stress in HCW contributing to burnout,^11^ with the critical drivers considered in two categories: internal (individual) and external (organisational).^12^ Individual factors include aspects of work-life balance, coping mechanisms and support structures.^3^ In addition, the individual factors of psychological flexibility (capacity to remain in the present with emotional awareness) and resiliency (ability to quickly adapt to stress and adversity) function as mediators in reducing the effects of stress and burnout.^13,14^ Organisational factors are the predominant contributor to burnout^15^ and encompass aspects of the work environment, including workload, resources, institutional culture and autonomy.^2^

The ED is a high-stress environment,^16^ often overcrowded,^17^ understaffed and poorly equipped in low- to middle-income countries (LMIC).^18^ In addition, the coronavirus disease 2019 (COVID-19) pandemic has aggravated existing health system strain causing high levels of psychosocial stress with unprecedented challenges in healthcare delivery and work conditions.^19^ Thus, intensifying the burden of burnout on ED staff.^6,20^

Concerns have emerged from various studies indicating rising levels of burnout associated with increasing costs and consequences to individuals and organisations.^12,21,22^ However, there are significant variations in the reported incidence of burnout between specialities and countries attributed to inconsistent burnout definitions, non-standardised assessment methods and differing thresholds across studies.^12,23^

Despite an international acceleration in burnout research among HCW due to the COVID-19 pandemic, there remains limited data on the prevalence of burnout in LMICs,^10,13^ especially in sub-Saharan Africa.^24,25^ In addition, South African research before the COVID-19 pandemic indicated high burnout levels in doctors^18,26^ and nurses,^27,28^ with no local studies assessing burnout in non-clinical staff.^29,30,31^ There is a need for burnout prevalence research during and after crises^32,33^ at an organisational level,^22^ with an emphasis on assessing the risk factors of burnout^3,24^ and effectiveness of intervention strategies^9,10^ to guide health system policy decisions.^32^ In addition, there is limited research evaluating staff perceptions of interventions and their effectiveness in South Africa.^9,10^

Burnout is a systems-level problem requiring effective strategies focusing on individual and structural (or organisational) solutions. In addition, the complexity of workplace factors contributing to burnout^3^ necessitates combined approaches.^34^ However, initiatives are often individual-focused, placing responsibility on HCW to develop coping mechanisms to improve resilience, neglecting organisational sources of chronic workplace stress beyond the HCW’ locus of control.^22^ The prevention of burnout in healthcare settings is a shared responsibility of healthcare systems, organisations and HCW.^2^ Furthermore, implementing structural reforms in healthcare organisations remains challenging in the South African context,^19^ but necessary for developing sustainable strategies to reduce burnout, ensure staff well-being and improve healthcare quality.

The aim of this research was to determine the prevalence of burnout in ED staff (doctors, nurses and non-clinical staff) at Tygerberg Hospital (TBH) via a validated survey using the Maslach Burnout Inventory Human Services Survey (MBI-HSS). In addition, the survey explored ED staff’s awareness and utilisation of intervention programmes to hypothesise leverage points to optimise the effectiveness of strategies. As burnout varies based on organisational context, understanding local burnout prevalence is essential to planning effective intervention strategies^12^ and facilitating change in organisations and the healthcare system.^35^

## Research methods and design

### Study design

This cross-sectional study measured burnout and intervention-related factors of ED staff working at TBH.

### Study setting

Tygerberg Hospital is an academic tertiary public facility in Cape Town, South Africa. The hospital consists of 1380 beds and serves a population of approximately 3.5 million people, predominantly from low-income areas and informal settlements. Tygerberg Hospital is the largest hospital in the Western Cape, receiving a high volume of acute complex cases. Emergency department services are provided by the internal medicine, emergency medicine, surgery and trauma departments across separate units (trauma, medical emergencies, surgical emergencies and resuscitation) within the hospital. Emergency department staff (doctors, nurses and non-clinical staff) work a minimum of 8–12-h shifts, with doctors exposed to shifts exceeding 24 h at least weekly. All staff are required to work unsocial hours, including weekend shifts, of varying frequency and duration each month. The hospital has one on-site counsellor to support staff with individual-focused initiatives for burnout through the Employee Health and Wellness Programme accessed by referral or self-presentation.

### Study population and sampling strategy

All staff (*N* = 227) employed in units providing ED services at TBH were eligible to participate in the study. Clinical and non-clinical staff were included in the target population as both groups are exposed to similar external factors contributing to burnout. The study population included 30 doctors (interns, medical officers, registrars and specialists), 150 nurses (nursing assistants, staff nurses, general professional nurses and specialist professional nurses), and 47 non-clinical staff (administration, porters, security and domestic workers). Staff employed as locums or for less than 1 month were excluded. A non-probabilistic convenience sampling strategy was utilised because of a time-dependent data collection period. A minimum sample size of 77 was targeted to achieve ideal power of 80% for research based on *a priori* power analysis at 5% significance, medium effect size (Cohen’s *d* = 0.15), and three predictors: emotional exhaustion (EE), depersonalisation (DP) and personal accomplishment (PA).^36,37^ In addition, a representative sample of doctors, nurses and non-clinical staff was targeted during different shifts and days of the week to reduce selection bias.

The study population was identified through the Manager of Medical Services at TBH, with full details of the study provided electronically to line managers of the emergency units. A cover letter was distributed to eligible staff via line managers in three languages (English, Afrikaans and isiXhosa), which explained the purpose of the study and access instructions to the electronic survey. The purpose was described as evaluating job-related attitudes to reduce participant sensitisation to burnout and response bias. Recruitment was via electronic communication, the cover letter, and an in-person hospital campaign utilising a portable device for survey completion to increase access to participants without compatible devices or language fluency. Participants were advised to complete the survey individually; however, technical assistance was permitted from line managers or the researcher. All survey responses were completed electronically. Informed consent was obtained on accessing the survey, and participant anonymity was ensured.

### Data collection

Data were collected via a structured self-administered electronic survey hosted on Qualtrics Core XM platform.^38^ The survey comprised four sections, namely, demographics, the MBI-HSS (MP), two close-ended questions on intervention awareness and utilisation, and two open-ended questions on recommendations to increase intervention uptake and suggested interventions to reduce burnout in staff. The demographics, close-ended and open-ended questions were created by a qualified medical doctor who has been practising in the emergency medicine field for 10 years and were assessed for face validity by an expert in the field of psychiatry and the Manager of Medical Services at TBH. A pilot study was conducted in July 2022 among five HCW to assess technological accessibility, flow and clarity of the survey with the wording of two questions refined for clarity. The study was conducted in October 2022.

The MBI is the gold standard in burnout measurement assessing the syndrome in the subscales of EE, DP and PA.^12,39^ The instrument was previously validated in South Africa for emergency medical services^40^ and nurses,^28^ with utilisation for burnout assessment of doctors in the ED setting.^18^ In addition, the MBI-HSS has been administered over a variety of countries, languages, cultural contexts and occupations with the validity confirmed through various studies and meta-analyses.^41,42,43,44^ Furthermore, the reliability was estimated using Cronbach’s alpha coefficients for EE (α = 0.89), DP (α = 0.77), and PA (α = 0.74).^41^ Permission to use the MBI instrument was obtained. The total scores for each subscale were divided into three tiers (low, moderate and high) based on established reference ranges (Table 1).^26,45^ Burnout was defined as high EE and high DP or high EE and low PA.^41,46^

**TABLE 1:** Maslach Burnout Inventory Human Services Survey subscale total score reference ranges.

Subscales	Emotional Exhaustion (EE)	Depersonalisation (DP)	Personal accomplishment (PA)
Low	0–16	0–6	≤ 31
Moderate	17–26	7–12	32–38
High	≥ 27	≥ 13	≥ 39

*Source:* Lim WY, Ong J, Ong S, et al. The Abbreviated Maslach Burnout Inventory can overestimate burnout: A study of anesthesiology residents. J Clin Med. 2019;9(1):61. https://doi.org/10.3390/jcm9010061

### Data analysis

Quantitative analyses were conducted using Statistical Package for the Social Sciences (SPSS) software version 28.0.1 for Mac and Microsoft Excel. All statistical tests were two-sided, and a 5% significance level (*p* < 0.05) was used. Means and standard deviations (s.d.) were calculated for continuous variables, and frequencies and percentages determined for categorical variables. Maslach Burnout Inventory Human Services Survey scale scores were calculated separately and aggregated to determine the sample’s burnout levels and prevalence.

The internal consistency of the study was determined by calculating Cronbach’s alpha coefficient. One-way analysis of variance (ANOVA) and least-significant difference (LSD) post hoc analyses were used to compare the MBI-HSS scores with demographic and intervention variables. Categorical variables were compared using chi-square analyses and Fisher’s exact test. Pearson correlation was calculated for continuous variables to determine linear associations.

Qualitative data from open-ended intervention questions were analysed using thematic analysis^47^ employing ATLAS.ti Mac version 22.1.0, a computer-assisted qualitative data analysis software. The first phase involved reading responses to become familiar with the content and develop an overview of the data. Thereafter, an inductive approach was used to identify segments of meaning through level 1 open-coding with level 2 free-coding refining the factors. Conceptual analysis involved categorising factors into groups and determining the relationships between groups to identify themes. The final phase involved reviewing the literature, research questions, themes, groups and factors to guide the development of the conceptual framework. Quantitative analysis of the frequency occurrences of factors determined the list order and prominence of groups and themes utilised in the framework.^47,48^ Primary analysis was performed by a qualified doctor practising in the emergency medicine field outside South Africa, and independent to the facility under investigation with an interest in mental health awareness. The qualitative data analysis process, coding and themes were audited for accuracy, reliability and validity by an independent researcher with a background in neuroscience and an expert in the fields of psychiatry and leadership development.

### Ethical considerations

The study was approved by the Social, Behavioural, and Education Research Ethics Committee of the University of Stellenbosch (REC: SBE 25406) and permission to conduct the study at TBH from the National Health Research Database (reference: WC_202209_024). A protocol for potential emotional discomfort was included in the survey with contact information provided for counselling and support services.

## Results

### Demographic characteristics of the sample

A total of 109 ED staff participated in the survey (*N* = 227). Incomplete surveys (*n* = 7) were removed with 102 valid surveys (effective response rate = 44.93%) contributing to the final analysis: consisting of 19 doctors (18.63%), 56 nurses (54.90%) and 27 non-clinical staff (26.47%).

The participants were between the ages of 22 and 59 years (*M* = 37.28, s.d. = 8.99), and the majority were female (72.55%) and unmarried (60.78%). Nursing formed the dominant staff group (54.90%), with a substantial proportion of participants working in the trauma department (39.22%) as depicted in Table 2. The mean employment history since completing education was 10.54 ± 8.93 years with the average employment at TBH 6.55 ± 7.69 years.

**TABLE 2:** Analysis of burnout prevalence and demographic variables.

Variables	*N*	%	Non-burnout		Burnout		*p*	*X* ^2^
			*n*	%	*n*	%		
**Gender**	-	-	-	-	-	-	0.87	0.028
Female	74	72.55	41	55.41	33	44.59	-	-
Male	28	27.45	15	53.57	13	46.43	-	-
Total	102	100	56	54.90	46	45.10	-	-
**Language**	-	-	-	-	-	-	1.00†	-
Afrikaans	28	27.45	15	53.57	13	46.43	-	-
English	32	31.37	18	56.25	14	43.75	-	-
Xhosa	38	37.25	20	52.63	18	47.37	-	-
**Education**	-	-	-	-	-	-	0.41	6.114
Primary school	1	0.98	1	100.00	0	-	-	-
High school	35	34.31	23	65.71	12	34.29	-	-
Diploma	24	23.53	12	50.00	12	50.00	-	-
Bachelor’s degree	26	25.49	12	46.15	14	53.85	-	-
Honour’s degree	3	2.94	2	66.67	1	33.33	-	-
Post-graduate diploma	11	10.78	6	54.55	5	45.45	-	-
Master’s degree	2	1.96	0	-	2	100.00	-	-
**Marital Status**	-	-	-	-	-	-	0.46†	-
Divorced or separated	4	3.92	1	25.00	3	75.00	-	-
Married	36	35.29	19	52.76	17	47.22	-	-
Single	62	60.78	36	58.06	26	41.94	-	-
**Staff groups**	-	-	-	-	-	-	< 0.05*	6.020
Doctors	19	18.63	8	42.11	11	57.89	0.06†	-
Interns	12	11.76	4	33.33	8	66.67	-	-
Medical officer	3	2.94	3	100.00	0	-	-	-
Registrar	4	3.92	1	25.00	3	75.00	-	-
Non-clinical staff	27	26.47	20	74.07	7	25.93	0.06†	-
Administration	3	2.94	2	66.67	1	33.33	-	-
Domestic service staff	9	8.82	8	88.89	1	11.11	-	-
Porter	7	6.86	6	85.71	1	14.29	-	-
Security	8	7.84	4	50.00	4	50.00	-	-
Nursing	56	54.90	28	50.00	28	50.00	0.06†	-
Nursing assistant	20	19.61	12	60.00	8	40.00	-	-
Staff nurse	9	8.82	6	66.67	3	33.33	-	-
General professional nurse	12	11.76	5	41.67	7	58.33	-	-
Specialist professional nurse	15	14.71	5	33.33	10	66.67	-	-
**Department**	-	-	-	-	-	-	0.12†	-
Internal medicine	26	25.49	13	50.00	13	50.00	-	-
Surgery	29	28.43	12	41.38	17	58.62	-	-
Trauma	40	39.22	25	62.50	15	37.50	-	-

* , *p* < 0.05.

† , Fisher exact test.

The internal consistency estimated by Cronbach’s alpha was determined for EE (α = 0.89, 95% confidence interval [CI]: 0.85, 0.91), DP (α = 0.69, 95% CI: 0.58, 0.77) and PA (α = 0.78, 95% CI: 0.67, 0.85) indicating adequate reliability for the study.

### Burnout levels and prevalence estimates

In all, 46 participants met the criteria for burnout (i.e. burnout defined by high EE and high DP or high EE and low PA), indicating a prevalence of 45.10% (95% CI: 35.39, 54.80) in the sample. In addition, 73 participants (71.57%) experienced high scores in EE or DP. There were 15 participants (14.71%) who experienced severe burnout across all three subscales (i.e. high EE, high DP, and low PA). Of interest, two-thirds of participants (66.67%) displayed moderate to high levels of burnout in all three subscales. In contrast, only nine participants (8.82%) were identified with low risk for burnout across all subscales and were considered engaged staff (i.e. low EE, low DP, and high PA).

The mean scores of the MBI-HSS subscales showed high EE (*M* = 28.27, s.d. = 28.27), moderate DP (*M* = 11.06, s.d. = 7.37) and moderate PA (*M* = 35.82, s.d. = 9.48) suggesting EE as the predominant characteristic of burnout in the sample. The results of ANOVA shown in Table 3 revealed high EE scores were most significant in nursing (*F* = 5.010, *p* < 0.01), while high DP scores were most significant in doctors (*F* = 5.961, *p* < 0.01). In addition, non-clinical staff scored significantly lower across all the MBI-HSS subscales of EE (*F* = 5.010, *p* < 0.01), DP (*F* = 5.961, *p* < 0.01), and PA (*F* = 6.186, *p* < 0.01). Furthermore, bivariate analysis of correlation coefficients revealed that the level of education had a weak positive linear association with EE (*r* = 0.23, *p* = 0.02) and DP (*r* = 0.21, *p* = 0.01) scores. At the same time, PA had a weak negative association with the level of education (*r* = −0.25, *p* = 0.01). Therefore, indicating higher degrees of EE and DP, with increasing levels of education but lower PA.

**TABLE 3:** Analysis of variance analysis of demographic variables and Maslach Burnout Inventory Human Services Survey subscale scores.

Variables	Emotional exhaustion (EE)		Depersonalisation (DP)		Personal accomplishment (PA)	
	Mean ± s.d.	*p*	Mean ± s.d.	*p*	Mean ± s.d.	*p*
**Gender**	-	0.04*	-	0.52	-	0.74
Female	30.03 ± 13.85	-	11.35 ± 7.52	-	36.01 ± 9.66	-
Male	23.64 ± 13.21	-	10.29 ± 7.02	-	35.32 ± 9.15	-
**Language** ‡	-	0.35	-	0.53	-	0.02*
Afrikaans	25.14 ± 15.72	-	10.11 ± 8.33	-	32.96 ± 9.42	-
English	30 ± 14	-	12.25 ± 6.59	-	35.59 ± 6.91	-
Xhosa	29.42 ± 12.68	-	11.03 ± 7.41	-	39.24 ± 9.83	-
**Education§**	-	0.10†	-	0.07†	-	0.01*†
Primary School	18 ± 0	-	11 ± 0	-	48 ± 0	-
High School	23.11 ± 13.38	-	8.37 ± 6.65	-	38.63 ± 10.65	-
Diploma	30.38 ± 13.83	-	11.21 ± 7.65	-	35.33 ± 8.40	-
Bachelor’s degree	32.62 ± 14.36	-	14.73 ± 7.54	-	33.46 ± 7.82	-
Honour’s degree	24 ± 6.56	-	9.67 ± 4.93	-	39.67 ± 4.62	-
Post-graduate diploma	29.64 ± 13.34	-	9.91 ± 6.28	-	33.64 ± 10.10	-
Master’s degree	41 ± 2.83	-	17 ± 8.49	-	23.50 ± 6.37	-
**Marital Status¶**	-	0.86	-	0.71	-	0.27
Divorced or separated	29.75 ± 18.89	-	12.5 ± 6.14	-	30 ± 6.98	-
Married	29.14 ± 13.68	-	11.72 ± 6.91	-	34.78 ± 7.86	-
Single	27.68 ± 13.95	-	10.58 ± 7.75	-	36.81 ± 10.35	-
**Staff Groups**	-	< 0.01*	-	< 0.01	-	< 0.01*
Doctors	30.21 ± 13.48	-	14.26 ± 6.51	-	31.58 ± 6.7	-
Non-clinical staff	21.3 ± 12.38	-	7.37 ± 5.81	-	40.63 ± 8.71	-
Nursing	30.98 ± 13.81	-	11.75 ± 7.69	-	34.95 ± 9.81	-
**Department**	-	< 0.01*	-	< 0.01*		0.66
Internal medicine	30.31 ± 12.33	-	12.5 ± 7.66	-	36.04 ± 9.69	-
Surgery	35.14 ± 10.25	-	13.21 ± 6.93	-	34.66 ± 8.58	-
Trauma	24.5 ± 14.75	-	9.93 ± 7.11	-	35.85 ± 10.17	-

s.d., standard deviation; vs, versus; EE, emotional exhaustion; DP, depersonalisation; PA, personal accomplishment.

* , *p* < 0.05.

† , Kruskal-Wallis Test;

‡ , Post-hoc test (*p*): Xhosa vs. Afrikaans (< 0.01);

§ , Post-hoc test (*p*): Master’s degree vs. high school (0.02); Master’s degree vs. primary school (0.02); Bachelor’s degree vs. high school (< 0.01);

¶ , Post-hoc test (*p*): Emotional exhaustion post-hoc test (*p*): Doctors vs. non-clinical (0.03); Nursing vs. non-clinical (< 0.01).

Table 4 depicts the relationship between the demographic variables and burnout levels among ED staff. Interns were associated with significant levels of burnout across all subscales: high EE (*X*^*2*^ = 45.733, *p* < 0.01), high DP (*p* < 0.01) and low PA (*X*^*2*^ = 50.388, *p* < 0.01). In addition, specialist professional nurses were associated with high levels of EE (*X*^*2*^ = 45.733, *p* < 0.01) and DP (*p* < 0.01). Of interest, doctors were significantly associated with low PA (*X*^*2*^ = 14.483, *p* < 0.01), whereas domestic service staff were significantly associated with high PA (*X*^*2*^ = 50.388, *p* < 0.01) compared to other staff groups.

**TABLE 4:** Analysis of burnout levels and demographic variables.

Variables	Emotional exhaustion (EE)							Depersonalisation (DP)								Personal accomplishment (PA)								
	Low		Moderate		High		*p*	*X* ^2^	Low		Moderate		High		*p*	*X* ^2^	Low		Moderate		High		*p*	*X* ^2^
	*n*	%	*n*	%	*n*	%			*n*	%	*n*	%	*n*	%			*n*	%	*n*	%	*n*	%		
**Gender**	-	-	-	-	-	-	0.43	1.705	-	-	-	-	-	-	0.93	0.145	-	-	-	-	-	-	-	1.015
Female	17	22.97	12	16.22	45	60.81	-	-	24	32.43	18	24.32	32	43.24	-	-	23	31.08	15	20.27	36	48.65	-	-
Male	10	35.71	4	14.29	14	50.00	-	-	10	35.71	7	25.00	11	39.29	-	-	9	32.14	8	28.57	11	39.29	-	-
Total	27	26.47	16	15.69	57.84	59.00	-	-	34	33.33	25	24.51	43	42.16	-	-	31	31.37	23	22.55	47	46.08	-	-
**Language**	-	-	-	-	-	-	0.21†	-	-	-	-	-	-	-	0.02†	-	-	-	-	-	-	-	0.01*†	
Afrikaans	11	39.29	3	10.71	14	50.00	-	-	13	46.43	3	10.71	12	42.86	-	-	12	42.86	8	28.57	8	28.57	-	-
English	7	21.88	6	18.75	19	59.38	-	-	6	18.75	14	43.75	12	37.50	-	-	10	31.25	10	31.25	12	37.50	-	-
Xhosa	8	21.05	5	13.16	25	65.79	-	-	14	36.84	6	15.79	18	47.37	-	-	8	21.05	4	10.53	26	68.42	-	-
**Education**	-	-	-	-	-	-	0.32†	-	-	-	-	-	-	-	0.584†	-	-	-	-	-	-	-	0.19†	
Primary School	0	-	1	100.00	0	-	-	-	0	-	1	100.00	0	-	-	-	0	-	0	-	1	100.00	-	-
High School	13	37.14	4	11.43	18	51.43	-	-	17	48.57	7	20.00	11	31.43	-	-	8	22.86	5	14.29	22	62.86	-	-
Diploma	6	25.00	5	20.83	13	54.17	-	-	7	29.17	6	25.00	11	45.83	-	-	8	33.33	5	20.83	11	45.83	-	-
Bachelor’s Degree	5	19.23	3	11.54	18	69.23	-	-	6	23.08	6	23.08	14	53.85	-	-	10	38.46	8	30.77	8	30.77	-	-
Honour’s Degree	0		2	66.67	1	33.33	-	-	1	33.33	1	33.33	1	33.33	-	-	0		2	66.67	1	33.33	-	-
Post-graduate diploma	3	27.27	1	9.10	7	63.64	-	-	3	27.27	3	27.27	5	45.45	-	-	4	36.36	3	27.27	4	36.36	-	-
Master’s degree	0	-	0	-	2	100.00	-	-	0	-	1	50.00	1	50.00	-	-	2	100.00	0	-	0	-	-	-
**Marital Status**	-	-	-	-	-	-	0.84†	-	-	-	-	-	-	-	0.90†	-	-	-	-	-	-	-	0.38†	
Divorced or separated	1	25.00	0	-	3	75.00	-	-	1	25.00	1	25.00	2	50.00	-	-	3	75.00	0	-	1	25.00	-	-
Married	10	27.78	4	11.11	22	61.11	-	-	10	27.78	10	27.78	16	44.44	-	-	13	36.11	8	22.22	15	41.67	-	-
Single	16	25.81	12	19.35	34	54.84	-	-	23	37.10	14	22.58	25	40.32	-	-	16	25.81	15	24.19	31	50.00	-	-
**Staff Groups**	-	-	-	-	-	-	0.17†	-	-	-	-	-	-	-	0.04*	10.367	-	-	-	-	-	-	< 0.01*	14.483
Doctors	4	21.05	2	10.53	13	68.42	< 0.01*	45.733	3	15.79	6	31.58	10	52.63	0.02*†	-	9	47.37	7	36.84	3	15.79	< 0.01*	50.388
Interns	0		2	16.67	10	83.33	-	-	0	-	5	41.67	7	58.33	-	-	6	50.00	6	50.00	0	-	-	-
Medical Officer	3	100.00	0	-	0	-	-	-	2	66.67	1	33.33	0	-	-	-	2	66.67	0	-	1	33.33	-	-
Registrar	1	25.00	0	-	3	75.00	-	-	1	25.00	0	-	3	75.00	-	-	1	25.00	1	25.00	2	50.00	-	-
Non-clinical staff	12	44.44	3	11.11	12	44.44	< 0.01*	45.733	15	55.56	6	22.22	6	22.22	< 0.01*†		5	18.52	3	11.11	19	70.37	< 0.01*	50.388
Administration	2	66.67	0	-	1	33.33	-	-	1	33.33	1	33.33	1	33.33	-	-	1	33.33	2	66.67	0	-	-	-
Domestic service staff	1	11.11	3	33.33	5	5.56	-	-	6	66.67	2	22.22	1	11.11	-	-	0	-	0	-	9	100.00	-	
Porter	6	85.71	0	-	1	14.29	-	-	5	71.43	2	28.57	0	-	-	-	2	28.57	1	14.29	4	57.14	-	-
Security	3	37.50	0	-	5	62.50	-	-	3	37.50	1	12.50	4	50.00	-	-	2	25.00	0	-	6	75.00	-	-
Nursing	11	19.64	11	19.64	34	60.71	< 0.01*	45.733	16	28.57	13	23.21	27	48.21	< 0.01*†		18	32.14	13	23.21	25	44.64	< 0.01*	50.388
Nursing Assistant	4	20.00	5	25.00	11	55.00	-	-	5	25.00	6	30.00	9	45.00	-	-	8	40.00	2	10.00	10	50.00	-	-
Staff nurse	4	44.00	2	22.00	3	33.00	-	-	5	55.56	1	11.11	3	33.33	-	-	1	11.11	5	55.56	3	33.33	-	-
General professional nurse	1	8.33	3	25.00	8	66.67	-	-	5	41.67	1	8.33	6	50.00	-	-	3	25.00	2	16.67	7	58.33	-	-
Specialist professional nurse	2	13.33	1	6.67	12	80.00	-	-	1	6.67	5	33.33	9	60.00	-	-	6	40.00	4	26.67	5	33.33	-	-
**Department**	-	-	-	-	-	-	< 0.01*†	-	-	-	-	-	-	-	0.02*†	-	-	-	-	-	-	-	0.76†	-
Internal medicine	5	19.23	3	11.54	18	69.23	-	-	6	23.08	7	26.92	13	50.00	-	-	7	26.92	7	26.92	12	46.15	-	-
Surgery	2	6.90	4	13.79	23	79.31	-	-	5	17.24	8	27.59	16	55.17	-	-	9	31.03	9	31.03	11	37.93	-	-
Trauma	15	37.50	8	20.00	17	42.50	-	-	17	42.50	9	22.50	14	35.00	-	-	14	35.00	6	15.00	20	50.00	-	-

* , *p* < 0.05.

† , Fisher exact test.

The prevalence of burnout by demographic variables is quantified in Table 2. Burnout was significantly associated with staff groups (*X*^2^ = 6.020, *p* < 0.05) with findings of higher burnout in doctors and nursing staff, while lower burnout was observed in non-clinical staff, reinforcing the results of ANOVA indicating intergroup differences in MBI-HSS subscale scores. Furthermore, among staff groups the prevalence of burnout in doctors was 57.89% (*n* = 11), non-clinical staff 25.93% (*n* = 7) and nursing staff 50% (*n* = 28).

### Analysis of intervention insights

The overall awareness of existing interventions at TBH among participants was 41.18% (*n* = 42) with only 36.96% of participants (*n* = 17) experiencing burnout indicated being aware of programmes addressing burnout or stress. In addition, the utilisation of existing interventions among participants was low at 8.82% (*n* = 9), with only 8.70% (*n* = 4) of participants experiencing burnout accessing programmes addressing burnout or stress. Of interest, 88.89% of participants accessing interventions possessed a diploma or lower levels of education with a trend suggesting a weak negative correlation between intervention utilisation and level of education (*r* = −0.195, *p* = 0.05). Furthermore, intervention utilisation had a weak positive correlation to the number of years employed since qualification (*r* = 0.222, *p* = 0.03) and work experience at TBH (*r* = 0.289, *p* < 0.01).

**FIGURE 1:**
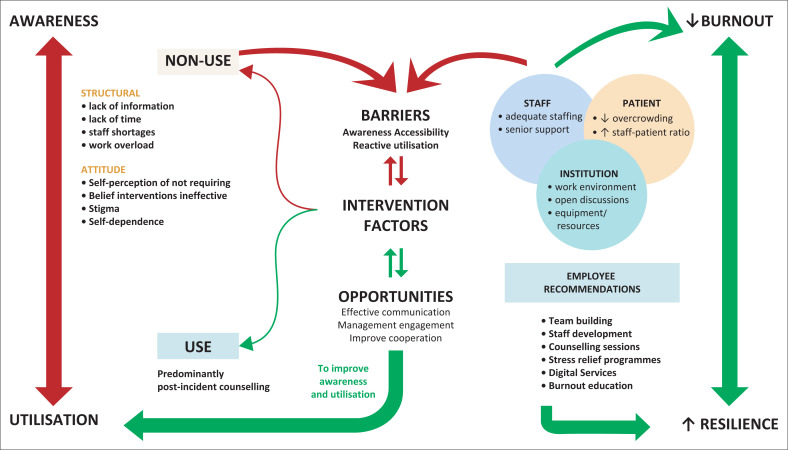
Intervention relationship model.

Figure 1 depicts the intervention-related factors, groups and themes identified through thematic analysis of qualitative data.^47^ The arrows depict proposed interactions between factors as ‘barriers’ and ‘opportunities’ influencing ‘burnout’, ‘resilience’, and intervention ‘awareness’ and ‘utilisation’ among ED staff.

Based on the frequency of occurrences of factors determined by thematic analysis, most participants were unaware of existing interventions at TBH because of ‘structural’ and ‘attitude’ themes. ‘Lack of information’ was identified as the main factor (58%) for the reasons of ‘non-use’. In addition, more than half the participants (54.12%) who reported ‘lack of information’ and a third of participants (36.36%) with ‘self-perception of not requiring’ interventions were experiencing burnout. The predominant ‘use’ case for accessing interventions was referrals for ‘post-incident counselling’ (44%), with only one participant having utilised the programme for burnout. The findings indicate the main ‘barriers’ to intervention utilisation were ‘awareness’, ‘accessibility’, and ‘reactive utilisation’ (the use of services only following an incident as opposed to pre-emptive or prophylactic engagement with services). ‘Staff’, ‘patient’ and ‘institution’-related themes were prominently reported in participant recommendations to reduce the levels of ‘burnout’. The three themes are interrelated and influence structural factors representing potential ‘opportunities’ to reduce burnout or ‘barriers’ if left unresolved. In addition, the ‘opportunities’ of ‘effective communication’, ‘management engagement’ and ‘improve cooperation’ were identified from suggestions to raise ‘awareness’ and improve intervention ‘utilisation’. Finally, the specific ‘staff recommendations’ identified provide the potential to enhance ‘resilience’ in ED staff thereby mediating the effects of burnout.

## Discussion

The key finding of 45.1% burnout prevalence is marginally lower than global estimates for ED staff during the COVID-19 pandemic.^9^ However, the result is comparable to prevalence estimates before the pandemic,^49,50^ and for sub-Saharan Africa of 40.0% to 80.0%.^24^ In addition, the results of estimated prevalence for staff groups (doctors, nursing and non-clinical staff) exceed findings from international studies in the ED setting.^29,51,52^ Maslach et al.^41^ described engagement as the state opposed to burnout, characterised by low EE, low DP and high PA. In this study, only 8.82% of ED staff experienced engagement with their work. Therefore, it is evident across all ED staff groups that the burden of burnout at TBH is significant. Burnout is a health system challenge that undermines the ability of HCW to deliver safe, high-quality care and negatively impacts well-being.^53^

Among doctors, this study found marginal differences in mean EE (30.21 vs. 30.5) and DP (14.26 vs. 14.6) scores compared to the mean scores of doctors in Western Cape district hospitals.^15^ However, the mean scores for PA (31.58 vs. 34.1) were notably lower in the TBH study sample,^15^ with the prevalence of EE greater than other studies in this setting.^15,18^ This result suggests doctors in the study sample are experiencing high levels of EE and lower levels of PA from their work compared to prior South African studies. In addition, this study found DP to be significantly higher in doctors than in other staff groups. Local studies have identified high subscale scores for DP among South African doctors,^15,18^ while this study’s finding of significance among interns is particularly concerning because of the presence of callus and dehumanised perceptions of patients at an early stage of their medical careers. Furthermore, the significant finding of high burnout levels across all three subscales (high EE, high DP and low PA) contributed to a significantly high burnout prevalence (66.7%) in interns. These findings indicate interns working in ED are severely overextended and exhausted by their work, impacting patient care through impersonal interactions and well-being through diminished self-efficacy.^41^

Among nursing staff, the prevalence of burnout was found to be lower than doctors, consistent with international studies^54^ but contrary to other studies from sub-Saharan Africa.^24^ In addition, this study found high mean EE (30.98 vs. 22.15) and DP (11.75 vs. 7.22) scores with similar PA (34.95 vs. 34.5) scores to a national survey of nurses.^28^ The comparison suggests nurses in the study sample are experiencing higher levels of EE associated with a greater degree of dehumanised patient interactions than the national baseline. Furthermore, specialist professional nurses were found to experience significantly high levels of EE and DP associated with increased burnout among staff groups in the study. Work overload, interpersonal conflict and organisational constraints are identified as predictors for high EE and DP in nurses with discordant patient relationships, impacting patient care.^55^ In addition, high EE and DP are associated with lower levels of job satisfaction and an intention to leave the workplace in nurses.^28,56^ Specialist professional nurses face the overwhelming challenge, characteristic of public sector hospitals in South Africa, of managing high patient volumes with low nurse ratios.^17^ Such emotionally demanding tasks influence nurse-patient interactions increasing emotional dissonance in nurses. Thus, their well-being is affected, which leads to burnout with reduced productivity compromising quality of care.^57^

Among non-clinical staff, the findings of this study represent novel data utilising the MBI-HSS in a hospital setting with staff groups (administration, domestic service staff, porters and security) not typically assessed in existing burnout research. Organisational factors relating to the work environment and workload are the predominant factors associated with the development of burnout in HCW,^3,15^ with an expectation for a similar impact in non-clinical staff working in hospitals with patients. The findings of this study indicate that non-clinical staff experienced significantly lower burnout across all MBI-HSS subscales compared to doctors and nurses. There are limited published data for comparison, with few studies comparing burnout among clinical and non-clinical staff with conflicting findings and only encompassing administration staff.^29,31^ Despite lower burnout levels in non-clinical staff, the proportion of high EE (44.44%) within the staff group is substantial, indicating an impact associated with work environment factors in the ED of patient volumes, insufficient resources, and understaffing.^18,24^

The finding of high EE and DP within the sample compared to normative studies^41,51^ reinforces concern for broader health system challenges negatively impacting the quality of care with ED staff facing significant overextension impacting well-being and acrimonious interactions compromising patient care because of the work environment.^15,26,55^ The rising prevalence of burnout in health systems is not sustainable. Healthcare leaders and policymakers need to prioritise the system-level changes necessary to reduce burnout, thereby improving staff well-being and patient care.^53^ The existing interventions at TBH are individual-focused, placing responsibility on ED staff to access resources to develop coping mechanisms or improve resilience.^2^ However, this study’s findings indicate alarmingly low intervention awareness and utilisation levels, with even poorer rates among participants experiencing burnout. In addition, the organisational sources of chronic workplace stress (work overload and staff shortages) inherently act as barriers to accessing interventions and are beyond employees’ level of control.^19^ Furthermore, the opportunities identified to enhance awareness and utilisation of interventions through effective communication, management engagement and improved cooperation call for a systematic approach at an organisational level.^22^ Therefore, burnout prevention in healthcare settings is a shared responsibility of healthcare systems, organisations and HCW.^34^

The high prevalence of burnout at TBH in ED staff (doctors, nurses and non-clinical staff) demonstrated by this study emphasises the need for greater engagement by hospital management and system-level decision-makers. There is a need for the development of cooperative intervention strategies and health workforce governance policies that are strategically aligned to the factors identified as driving burnout (structural, attitude, staff, patient, institutional) and existing barriers (awareness, accessibility, reactive utilisation) to interventions.

Lack of awareness and accessibility are barriers to intervention strategy effectiveness and were found to be prominent factors influencing utilisation among ED staff at TBH. Therefore, reliance on existing individual-focused interventions at a system level (national programme for government employees) requires redress by organisational-level management, reinforced by the poor utilisation rates among participants despite the prevalence of burnout symptoms. A critical starting point for hospital management is to address awareness of burnout and accessibility to existing interventions promoting utilisation as a pre-emptive measure through effective communication. The findings on intervention use, awareness and staff recommendations contribute to guiding decision-making and support the need for strategies to raise awareness, promote well-being and develop evidence-based organisational programmes to enhance coping mechanisms (resiliency) for burnout in HCW.

This study has contributed to understanding the scope and estimated magnitude of the burden of burnout among ED staff at TBH. In addition, findings related to non-clinical staff provide novel data to health research in South Africa and important implications for including non-clinical staff in developing and designing intervention strategies at the organisational and system levels. Furthermore, the study contributes to the limited existing knowledge regarding the prevalence of burnout among ED staff in South Africa and provides evidence that burnout is rising. Thus, the need for system-level change by healthcare leaders and policymakers is emphasised to ensure the well-being of HCW and improve the quality of care in the South African healthcare system.

There were several limitations to this study. Firstly, the study was conducted at a single healthcare facility using convenience sampling, resulting in the under-representation of some staff occupations within the sample, limiting the generalisability of results to the population. Secondly, the study used an electronic survey only available in English with the potential for non-response error by systematically excluding some participants. Thirdly, despite undertaking a rigorous and systematic analysis of qualitative data using thematic analysis with independent auditing of coding and themes, the findings are potentially limited by respondent bias because of on-site interactions with participants during survey completion or researcher bias through unconscious positionality during analysis. Lastly, the study utilised the MBI-HSS for the assessment of burnout. There are significant variations in reporting results among published research in South Africa, with the potential for standardisation to improve the comparison of findings in future. In addition, there are limited validation studies assessing the MBI-HSS in South Africa, with further studies required on the psychometric validity and cultural adaptation of the standard and translated versions of the MBI-HSS in local languages to determine construct equivalence and improve utilisation in the South African context.

There is a lack of South African research on burnout in healthcare settings of large sample sizes and comparing burnout between HCW, facilities, sectors and provinces. Therefore, future research should focus on study designs to enable extensive population sampling and screening, providing evidence of the nature and prevalence of burnout in the South African healthcare system. In addition, it is recommended that future research explore the effectiveness of intervention strategies in the South African context to guide policy design enabling system-level change. Furthermore, additional studies are required to evaluate the nature and prevalence of burnout in non-clinical staff.

## Conclusion

There was a high prevalence of burnout among ED staff at TBH, with evidence of the burden in this setting worsening. Doctors and nurses were affected more than non-clinical staff, with interns and specialist professional nurses identified as risk groups. The solution to HCW burnout requires collaborative efforts at health system and organisational levels to develop effective intervention strategies, inclusive of non-clinical staff. Organisational intervention strategies should prioritise addressing awareness and accessibility in the short term to improve utilisation. However, long-term goals must include management engagement in tackling system-level barriers in combination with initiatives that enhance staff resilience.
